# Influence of design features and brand of reverse shoulder arthroplasties on survivorship and reasons for revision surgery: results of 5,494 arthroplasties with up to 15 years’ follow-up reported to the Norwegian Arthroplasty Register 2007–2022

**DOI:** 10.2340/17453674.2024.41344

**Published:** 2024-08-27

**Authors:** Randi M HOLE, Anne Marie FENSTAD, Jan-Erik GJERTSEN, Geir HALLAN, Ove N FURNES

**Affiliations:** 1 The Norwegian Arthroplasty Register, Department of Orthopedic Surgery, Haukeland University Hospital, Bergen; 2 Department of Clinical Medicine, University of Bergen, Bergen, Norway

## Abstract

**Background and purpose:**

We aimed to report the survival of different reverse shoulder arthroplasty (RSA) designs and brands, and factors associated with revision. The secondary aim was to evaluate the reasons for revision.

**Methods:**

We included 4,696 inlay and 798 onlay RSAs reported to the Norwegian Arthroplasty Register (NAR) 2007–2022. Kaplan–Meier estimates of survivorship and Cox models adjusted for age, sex, diagnosis, implant design, humeral fixation, and previous surgery were investigated to assess revision risks. The reasons for revision were compared using competing risk analysis.

**Results:**

Overall, the 10-year survival rate was 94% (confidence interval [CI] 93–95). At 5 years all brands exceeded 90%. Compared with Delta Xtend (n = 3,865), Aequalis Ascend Flex (HR 2.8, CI 1.7–4.6), Aequalis Reversed II (HR 2.2, CI 1.2–4.2), SMR (HR 2.5, CI 1.3–4.7), and Promos (HR 2.2, CI 1.0–4.9) had increased risk of revision. Onlay and inlay RSAs had similar risk of revision (HR 1.2, CI 0.8–1.8). Instability and deep infection were the most frequent revision causes. Male sex (HR 2.3, CI 1.7–3.1), fracture sequelae (HR 3.1, CI 2.1–5.0), and fractures operated on with uncemented humeral stems had increased risk of revision (HR 3.5, CI 1.6–7.3).

**Conclusion:**

We found similar risk of revision with inlay and onlay designs. Some prosthesis brands had a higher rate of revision than the most common implant, but numbers were low.

The use of reverse shoulder arthroplasty (RSA) has increased [1,2] and new brands have emerged on the marked during the last 2 decades, which may introduce new types of complications.

The newer onlay design has shown some biomechanical advantages and supposedly a better range of motion than traditional inlay type implants [3], but reports on differences in survival are sparse [4].

The primary aim of our study was to evaluate the survival of different RSA designs and brands reported to the NAR from 2007–2022, and factors associated with revision. The secondary aim was to evaluate the reasons for revision in different RSA designs.

## Methods

This study was performed according to the Reporting of studies Conducted using the Observational Routinely collected health Data (RECORD) checklist.

This is a population-based (5.5 million inhabitants) national cohort study using data from the NAR. Shoulder arthroplasties have been registered in the NAR since 1994. The Delta III (DePuy Orthopaedics, Inc, Warsaw, IN, USA) was the only brand on the Norwegian market from 1994 until 2007. From 2007 several other brands were introduced [5].

Both primary operations and revisions are reported by the orthopedic surgeons, who complete a 1-page form directly after surgery. A completeness of reporting analysis has been conducted by combining the data in the NAR with data from the Norwegian Patient Register. The completeness of reporting of shoulder arthroplasties was 90.8% for primary operations and 84.6% for revisions in 2019–2020 [6], and 44 different hospitals have reported 1 or more RSAs in the study period. Several diagnoses could be given for each operation, and in cases with more than 1 diagnosis we chose 1 according to the hierarchy developed by the Nordic Arthroplasty Register Association (NARA) [7]. The brands included were classified as inlay or onlay according to the manufacturer’s description of the prosthesis in the surgical technique guides for each brand. Other features that influence the global lateralization were not included in the classification.

The NAR uses the unique personal identification number of each resident to link information from any subsequent revision to the primary surgery. A revision is defined as the insertion, exchange, or extraction of any of the prosthesis components. Several reasons for a revision could be given and, again, the hierarchy developed by NARA was used whenever more than 1 reason was given.

Information on death and emigrations was collected from the Norwegian National Population Register.

Outcomes were implant survival, risk of revision, and reasons for revision.

### Statistics

Descriptive statistics were used to give an overview of the patient demographics. Median time of follow-up for the different brands was estimated by the reverse Kaplan–Meier method. Implant survival with all revisions as endpoint was analyzed using the Kaplan–Meier method. Implant survival was reported at 5, 8, and 10 years. Results are presented with 95% confidence intervals (CI). Follow-up were censored at the time of revision, death, emigration, or end of study (December 31, 2022).

The revision risk for different implants and diagnoses was compared using Cox multiple regression analyses adjusted for age group, sex, diagnosis, humeral fixation, and previous surgery in the same shoulder. In cases with multiple revisions, only the time to the first implant failure was included in the analyses. We developed directed acyclic graphs (DAGs) for revision of RSA (see Appendix). The free program DAGitty (www.dagitty.net) version 3.1 (2023) was used to choose appropriate variables for the Cox models (Supplementary data). DAGs were developed individually for each exposure. The hazard ratio for the exposures (design, brand, and humeral fixation for each diagnosis) were adjusted according to this model. Hazard ratio (HR) was calculated at 5 years and for the whole study period. The 4 most common diagnoses were analyzed separately to identify risk factors and reasons for revision.

To test the proportional hazards assumption, we used the scaled Schoenfeld residuals on time in the “estat phtest command” in Stata (StataCorp LLC, College Station, TX, USA) and found the log hazard-function to be constant over time [8].

All tests were 2-sided and P < 0.05 was considered statistically significant.

In addition, competing risk analyses according to Fine and Gray were performed for the different brands and the most common primary diagnoses by calculating the subhazard ratios (SHR) [9] for each cause of revision. The reason to present the SHRs was to calculate correct estimates for revision for each cause separately. The SHRs describe the relative effect of potential covariates on the subdistribution hazard function. The endpoint was revision due to a specific cause with revision due to all other causes as the competing factor [10]. If the patient died or emigrated the follow-up time was censored.

The inlay group consisted mainly of Delta Xtend arthroplasties (82%). A sub-analysis with exclusion of the Delta Xtend was done when comparing inlay and onlay implants in the Cox regression analyses. The onlay design was introduced after the start of the study period, and a sub-analysis for the period where both onlay and inlay designs were used (2013–2022) was also done to ensure comparison in the same time period for the 2 designs.

All analyses were performed using the package SPSS statistics version 26.0.1.0 (IBM Corp, Armonk, NY, USA), the statistical package R Version 4.0.2 (R Foundation for Statistical Computing, Vienna, Austria), and Stata 17.0 (StataCorp LLC, College Station, TX, USA).

### Ethics, funding, data sharing, use of AI, and disclosures

The NAR has permission from the Norwegian Data Inspectorate to collect patient data based on written consent from the patients (ref 24.1.2017: 16/01622-3/CDG) and complies with the Norwegian and EU data-protection laws. The regulations of the Norwegian Data Protection Authority and the Norwegian personal protection laws prohibit the publication of the complete dataset. The Norwegian Arthroplasty Register is financed by the Western Norway health authorities. No AI-assisted technologies were used to produce this paper. RMH has received speaker fees from Ortomedic (Norwegian distributor for Depuy Synthes), Arthrex and Smith & Nephew, JEG and GH have received speaker fees from Ortomedic (Norwegian distributor for DePuy Synthes) and LINK Norway. OF has received fees for lectures on cementing technique for knee replacement given to Heraeus Medical and Ortomedic. Complete disclosure of interest forms according to ICMJE are available on the article page, doi: 10.2340/17453674.2024.41344.

## Results

**Figure 1:**
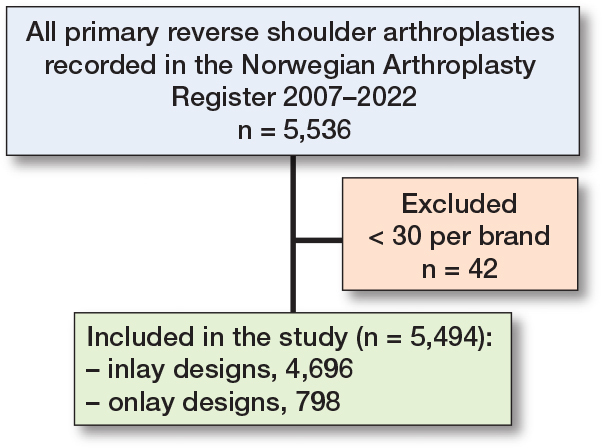
Flowchart of included and excluded reverse shoulder arthroplasties.

**Figure 2:**
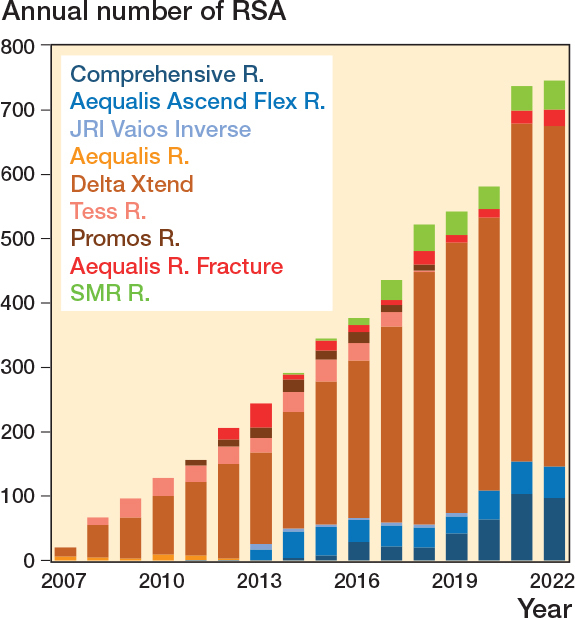
Number of reverse shoulder arthroplasties reported to the Norwegian Arthroplasty Register 2007–2022. Onlay designs in greenblue, inlay design arthroplasties in orange/red/brown.

Between 2007 and December 31, 2022, there were 5,536 RSAs registered in the NAR. After exclusion of brands with less than 30 arthroplasties performed (42 cases) the remaining 5,494 RSAs were included in the analyses (Figure 1). 4,696 were classified as inlay design, which is a traditional Grammont design where the humeral tray is seated within the metaphysis, and 798 as onlay design where the humeral tray sits on the metaphysis at the level of the humeral neck cut. All humeral implants were stemmed. Inlay design arthroplasties more often had a cemented humeral stem (77%) than onlay designs (22%). The annual number of RSAs increased from 20 in 2007 to 746 in 2022 (Figure 2). Onlay design arthroplasties were used from 2011 (Table 1). Delta Xtend was the most common brand; it was used in the entire study period and at 35 different hospitals. Some hospitals reported only 1 brand throughout the study period, while others had used several different brands.

**Table 1:** Included arthroplasties classified as either inlay or onlay of a reverse shoulder arthroplasty according to the position of the humeral tray as described by the manufacturer

Brand	n (%)	Hospitals	Design	Period used	Median F-U years (IQR)
Delta Xtend	3,865 (70)	35	Inlay	2007–2022	3.6 (1.7–6.0)
(DePuy Orthopaedics, Inc, Warsaw, IN, USA)					
Aequalis Reverse	32 (1)	3	Inlay	2007–2012	11.8 (9.1–13.6)
(Stryker GmbH, Selzach, Switzerland)					
TESS Reverse	261 (5)	6	Inlay	2008–2018	8.1 (6.1–10.9)
(Biomet, Warsaw, IN, USA)					
Promos Reverse	106 (2)	3	Inlay	2011–2018	6.5 (4.7–8.3)
(Smith&Nephew Orthop.AG, Zug, Switzerland)					
Aequalis Reverse II	190 (4)	5	Inlay	2012–2022	4.2 (1.6–7.9)
(Stryker GmbH, Selzach, Switzerland)					
SMR Reverse	242 (4)	4	Inlay	2014–2022	2.7 (1.2–4.2)
(Lima Corporate S.p.a, Udine, Italy)					
Comprehensive Reverse	392 (7)	10	Onlay	2011–2022	1.9 (1.0–3.3)
(Biomet, Warsaw, IN, USA)					
Aequalis Ascend Flex (Tornier Flex)	369 (7)	10	Onlay	2013–2022	3.9 (1.7–6.8)
(Stryker GmbH, Selzach, Switzerland)					
JRI Vaios Inverse	37 (1)	2	Onlay	2013–2019	5.7 (4.2–7.2)
(JRI Orthopaedics, Chapeltown, Sheffield, UK)					

F-U = follow-up; QR = interquartile range.

Mean age of the study population was 74 years (SD 9) in both groups, with 48% aged ≥ 75 and 37% aged 65–74 years at the time of surgery. 74% were women, with more women in the fracture group (83%) and the fracture sequelae group (81%) compared with the OA (71%) and rotator cuff arthropathy group (58%) (Table 2).

**Table 2:** Age and sex distribution within each diagnosis group of patients with a reverse shoulder arthroplasty. Values are count (%) unless otherwise specified

Diagnosis	Total	Women	Mean age (SD)	< 55	Age groups		≥ 75
					55–64	65–74	
Acute fracture	1,642 (30)	1,364 (83)	76 (8.6)	22 (1.3)	150 (9.1)	535 (33)	935 (57)
Primary osteoarthritis	1,349 (25)	955 (71)	74 (8.4)	29 (2.1)	137 (10)	490 (36)	693 (51)
Rotator cuff arthropathy	1,125 (21)	654 (58)	74 (7.8)	26 (2.3)	126 (11)	443 (39)	530 (47)
Fracture sequelae	817 (15)	662 (81)	72 (9.3)	45 (5.5)	143 (18)	335 (41)	294 (36)
Inflammatory arthritis	366 (6.7)	293 (80)	70 (9.8)	33 (9.0)	71 (19)	149 (41)	113 (31)
Instability sequelae	138 (2.5)	78 (57)	71 (10)	10 (7.6)	24 (18)	50 (36)	54 (39)
Other	56 (1.0)	32 (57)	66 (12)	10 (16)	15 (24)	18 (32)	13 (23)

Acute fracture was the most common reason for RSA (30%), followed by OA (25%), rotator cuff arthropathy (21%), and fracture sequelae (15%) (Table 3).

**Table 3:** Patient characteristics in reverse shoulder arthroplasties. Values are count (%) unless otherwise specified

Factor	Total	Inlay	Onlay
Arthroplasties	5,494	4,696 (85)	798 (15)
Women	4,038 (74)	3,472 (74)	566 (71)
Mean age at surgery (SD)	74 (8.9)	74 (8.9)	74 (8.6)
Age group			
< 55	175 (3.2)	150 (3.2)	25 (3.1)
55–64	666 (12)	564 (12)	102 (13)
65–74	2,021 (37)	1,740 (37)	281 (35)
≥ 75	2,632 (48)	2,242 (48)	390 (49)
Diagnosis			
Acute fracture	1,642 (30)	1,499 (32)	143 (18)
Primary osteoarthritis	1,349 (25)	1,037 (22)	312 (39)
Rotator cuff arthropathy	1,125 (21)	944 (20)	181 (23)
Fracture sequelae	817 (15)	740 (16)	77 (10)
Inflammatory arthritis	366 (6.7)	299 (6.4)	67 (8.4)
Instability sequelae	138 (2.5)	132 (2.8)	6 (0.8)
Other **^a^**	57(1.0)	45 (1.0)	12 (1.5)
Previous surgery in same			
shoulder **^b^**	1,054 (21)	916 (21)	138 (18)
Humeral fixation			
Cemented	3,763 (68)	3,591 (76)	172 (22)
Cementless	1,731 (32)	1,105 (24)	626 (78)
Duration of surgery in			
minutes, mean (SD) **^c^**	117 (37)	121 (37)	98 (30)
Follow-up, years			
median	3.6	3.8	2.6
IQR	1.7–6.3	1.8–6.5	1.2–5.4
min–max years	0–15	0–15	0–11

IQR = interquartile range.

a Other includes sequelae after infection, osteonecrosis, malignancy, dysplasia.

b Missing n = 437 (396 inlay, 41 onlay).

c Missing n = 209 (158 inlay, 51 onlay).

Irrespective of primary diagnosis, 188 (4.0%) cases with inlay designs and 42 (5.3%) with onlay designs were revised during the study period.

### Implant survival

**Figure 3:**
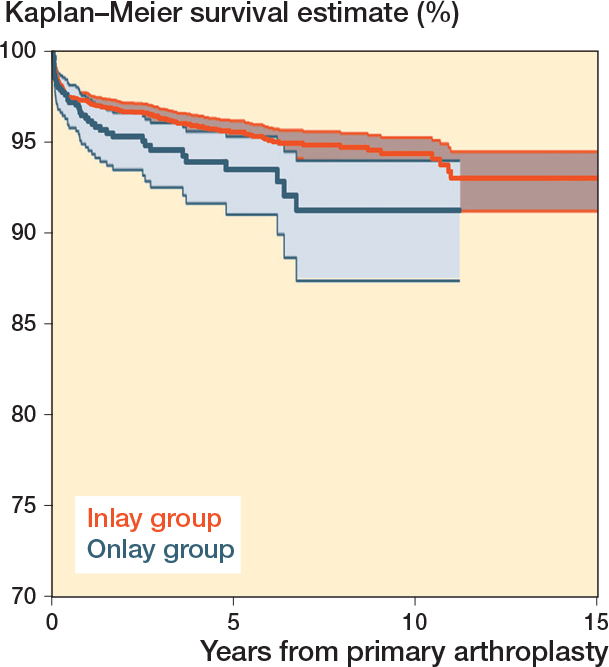
Kaplan–Meier survival for primary reverse shoulder arthroplasty by design (all diagnoses).

The 10-year survival for the primary RSA was 94% (CI 93–95). The 8-year survival was 95% (CI 94–96) for inlay design and 91% (CI 87–94) for onlay design (Table 4, Figure 3). All brands had 5-year survival of more than 90%, but only Delta Xtend and TESS had sufficient cases at risk to analyze the 10-year survival.

**Table 4:** Kaplan–Meier survival estimates (95%CI) at 5, 8, and 10 years, and Cox regression analysis at 5 years and for the entire period with adjustments for age group, sex, diagnosis, humeral fixation, and previous surgery for the included designs and brands of reverse shoulder arthroplasties. Endpoint all revisions

	At risk	5-year survival (CI)	5-year HR (CI)	At risk	8-year survival (CI)	At risk	10-year survival (CI)	HR all (CI)
Design								
Inlay	1,653	96 (94–96)	1 (Ref.)	723	95 (94–96)	364	95 (93–95)	1 (Ref.)
Onlay	210	94 (91–95)	1.3 (0.9–1.9)	52	91 (87–94)	3	–	1.2 (0.8–1.8)
Brand								
Delta Xtend	1,242	96 (96–97)	1 (Ref.)	501	96 (95–96)	250	95 (94–96)	1 (Ref.)
Aequalis Reversed	26	94 (77–98)	1.1 (0.2–7.9)	25	94 (77–98)	22	94 (77–98) **^a^**	0.8 (0.1–5.9)
TESS	217	95 (92–97)	1.4 (0.7–2.9)	130	93 (89–96)	74	92 (87–95)	1.7 (0.9–3.2)
Promos	67	93 (85–96)	1.9 (0.8–4.5)	30	91 (83–96)	12	91 (83–96) **^a^**	2.2 (1.0–4.9)
Aequalis Reversed II	72	92 (86–96)	2.5 (1.3–4.7)	42	92 (86–96)	1	–	2.2 (1.2–4.2)
SMR	35	93 (88–95)	2.3 (1.2–4.4)	1	–	1	–	2.5 (1.3–4.7)
Comprehensive	56	98 (96–99)	0.6 (0.3–1.5)	5	–	3	–	0.6 (0.3–1.5)
Aequalis Ascend Flex	135	93 (86–93)	2.6 (1.6–4.5)	39	88 (83–92)	1	–	2.8 (1.7–4.6)
JRI Vaios	19	91 (75–97)	2.6 (0.8–8.9)	10	85 (61–95)	1	–	2.7 (0.8–9.0)

a No revisions after 8 years.

**Figure 4:**
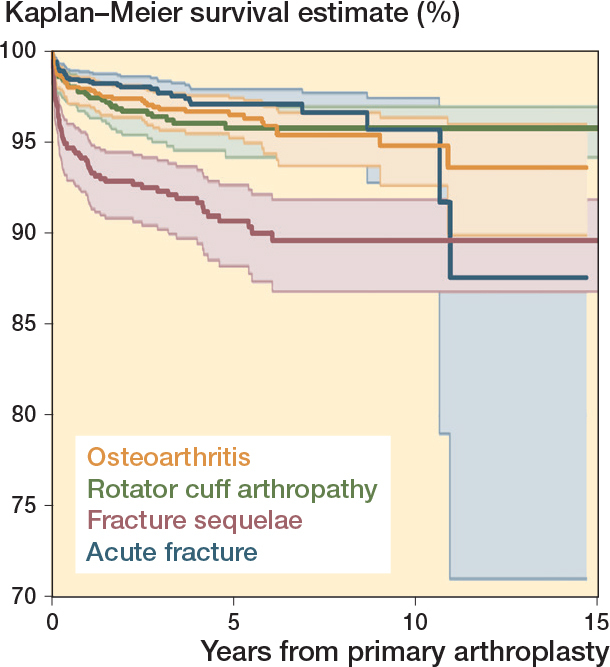
Kaplan–Meier survival curves for the 4 main primary indications for surgery.

The 10-year survival was 96% (CI 93–98) for acute fracture, 96% (CI 94–97) for rotator cuff arthropathy, and 95% (CI 93–96) for osteoarthritis. Fracture sequelae had the lowest 10-year survival of 90% (CI 87–92) (Figure 4).

**Figure 5:**
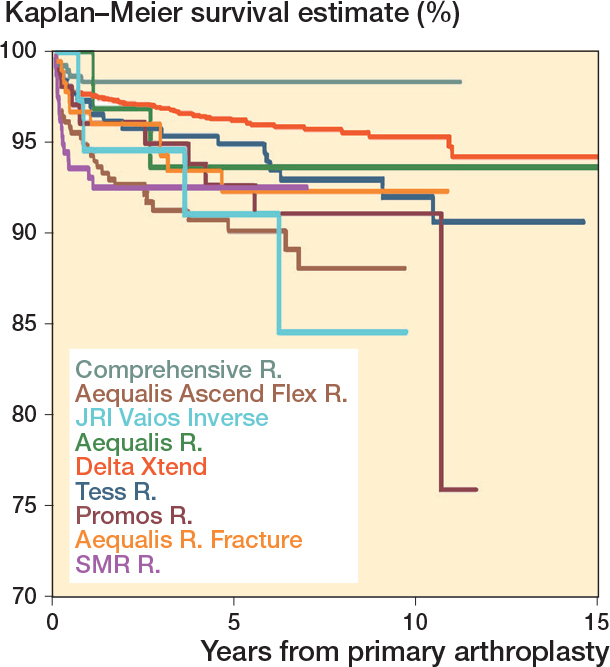
Kaplan–Meier survival curves for the arthroplasty brands (all diagnoses).

### Risk of revision

Onlay and inlay design had a comparable risk of revision (HR 1.2, CI 0.8–1.8) (Table 5). Sub-analysis with exclusion of Delta Xtend did not change this (HR 1.0, CI 0.6–1.5). Comparing the 2 designs in the period when both onlay and inlay design was used (2013–2022) also yielded similar results (HR 1.4, CI 0.9–2.1).

**Table 5:** Cox regression analyses to evaluate factors that can influence the risk for all-cause revision after reverse shoulder arthroplasty: age group, sex, primary diagnosis, implant design, humeral fixation, and previous surgery in the same shoulder

	HR (CI)	P
Design		
Inlay	1 (Ref.)	
Onlay	1.2 (0.8–1.8)	0.3
Sex		
Women	1 (Ref.)	
Men	2.3 (1.7–3.1)	< 0.001
Age group		
< 55	1.5 (0.8–2.7)	0.2
55–64	1.1 (0.7–1.7)	0.7
65–74	1.4 (1.0–1.9)	0.04
≥ 75	1 (Ref.)	
Humeral stem		
Cemented	1 (Ref.)	
Uncemented	1.7 (1.2–2.3)	0.002
Primary diagnosis		
Acute fracture	1 (Ref.)	
Fracture sequelae	2.7 (1.7–4.2)	< 0.001
Osteoarthritis	0.8 (0.5–1.3)	0.4
Rotator cuff arthropathy	0.8 (0.5–1.3)	0.4
Inflammatory arthritis	1.5 (0.8–2.5)	0.2
Instability sequelae	1.9 (0.9–4.0)	0.1
Other	2.4 (1.0–5.8)	0.06
Previous surgery		
No	1 (Ref.)	
Yes	1.1 (0.8–1.5)	0.5

Compared with the Delta Xtend, the Aequalis Ascend Flex (HR 2.8, CI 1.7–4.6), Aequalis Reversed II (HR 2.2, CI 1.2–4.2), SMR (HR 2.5, CI 1.3–4.7), and Promos (HR 2.2, CI 1.0–4.9) had an increased risk of revision for the whole follow-up period. With 5 years’ follow-up Aequalis Ascend Flex, Aequalis Reversed II, and SMR had increased risk of revision compared with Delta Xtend (Table 4).

Patients with fracture sequelae had increased risk of revision compared with acute fracture patients (HR 2.7, CI 1.7–4.2). Uncemented humeral stem (HR 1.7, CI 1.2–2.3) and male sex (HR 2.3, CI 1.7–3.1) were associated with a higher risk of revision. The increased risk for uncemented humeral stem was particularly evident for acute fracture diagnosis (HR 3.5, CI 1.6–7.3) (Table 6). Previous surgery to the shoulder did not increase the risk of revision in patients with fracture sequelae, rotator cuff arthropathy, or osteoarthritis.

**Table 6:** Cox regression analyses for the 4 main diagnoses with humeral stem fixation as exposure, adjusted for design, sex, age group, and previous surgery in the same shoulder for all-cause revision after reverse shoulder arthroplasty

	Acute fracture			Fracture sequelae			Rotator cuff arthropathy			Osteoarthritis		
	n	HR (CI)	P	n	HR (CI)	P	n	HR (CI)	P	n	HR (CI)	P
Humeral stem												
Cemented	1,482	1 (Ref.)		664	1 (Ref.)		668	1 (Ref.)		667	1 (Ref.)	
Uncemented	160	3.5 (1.6–7.3)	0.001	153	1.4 (0.8–2.6)	0.3	457	1.0 (0.4–2.2)	0.9	682	1.6 (0.8–3.3)	0.2
Design												
Inlay	1,499	1 (Ref.)		740	1 (Ref.)		944	1 (Ref.)		1,037	1 (Ref.)	
Onlay	143	1.4 (0.5–4.0)	0.5	77	0.8 (0.3–2.0)	0.6	181	2.4 (1.0–6.0)	0.06	312	1.1 (0.5–2.4)	0.8
Sex												
Women	1,364	1 (Ref.)		662	1 (Ref.)		654	1 (Ref.)		955	1 (Ref.)	
Men	278	1.6 (0.8–3.6)	0.2	155	2.3 (1.4–3.8)	0.002	471	3.2 (1.5–6.7)	0.002	394	2.3 (1.3–4.4)	0.007
Age group												
< 55	22	–	–	45	2.4 (1.0–6.1)	0.06	26	1.8 (0.2–15)	0.6	29	2.5 (0.5–12)	0.3
55–64	150	1.2 (0.4–3.6)	0.8	143	1.7 (0.8–3.7)	0.2	126	2.7 (1.0–7.3)	0.06	137	1.1 (0.3–3.9)	0.9
65–74	535	1.0 (0.5–2.1)	1.0	335	2.1 (1.1–3.8)	0.03	443	2.0 (0.9–4.5)	0.1	490	2.7 (1.3–5.4)	0.006
≥ 75	935	1 (Ref.)		294	1 (Ref.)		530	1 (Ref.)		693	1 (Ref.)	
Previous surgery												
No	1,435	1 (Ref.)		405	1 (Ref.)		675	1 (Ref.)		1,086	1 (Ref.)	
Yes	48	3.6 (1.1–12)	0.04	394	1.1 (0.7–1.7)	0.8	345	1.1 (0.6–2.3)	0.7	145	1.3 (0.6–3.0)	0.5

### Reasons for revision

Instability/dislocation was the most common reason for revision after RSA for acute fracture and fracture sequelae, while RSA for rotator cuff arthropathy was mainly revised due to infection or instability/dislocation, and RSA for osteoarthritis was mainly revised due to infection (Table 7). RSA due to fracture sequelae had increased risk of revision due to instability/dislocation (SHR 4.2 [2.1–7.7]) compared with RSA for acute fracture.

**Table 7:** Reasons for revision in n (% revised) of the 4 most common primary diagnoses for primary reverse shoulder arthroplasties by incidence and subhazard ratios (SHR) with 95% confidence interval (CI). All other revision causes were merged and treated as one competing risk in the analyses. Results are presented for the entire study period and adjusted for age group, sex, previous surgery, humeral fixation, and implant design. Acute fracture was used as reference where applicable. Other includes polyethylene wear, pain alone, mechanical problems (impingement, reduced mobility, malplacement of components)

Reason for revision	Acute fracture n = 1,642		Osteoarthritis n = 1,349		Rotator cuff arthropathy n = 1,125		Fracture sequelae n = 817	
	n (%)	SHR (CI)	n (%)	SHR (CI)	n (%)	SHR (CI)	n (%)	SHR (CI)
Instability	13 (0.8)	1 (Ref.)	10 (0.7)	0.5 (0.2–1.2)	11 (1.0)	0.6 (0.2–1.3)	29 (3.5)	3.7 (1.9–7.3)
Infection	12 (0.7)	1 (Ref.)	15 (1.1)	1.1 (0.5–2.4)	11 (1.0)	0.9 (0.4–2.0)	18 (2.2)	2.2 (1.0–4.8)
Fracture	8 (0.5)	1 (Ref.)	9 (0.7)	0.7 (0.2–1.9)	3 (0.3)	0.4 (0.1–1.5)	4 (0.5)	0.7 (0.2–2.6)
Glenoid loosening	–	–	5 (0.4)	1 (Ref.)	3 (0.3)	0.9 (0.2–3.5)	3 (0.4)	1.8 (0.4–9.2)
Humeral loosening	2 (0.1)	1 (Ref.)	–		–		3 (0.4)	3.0 (0.8–11.1)
Other	4 (0.2)	1 (Ref.)	7 (0.5)	1.6 (0.5–5.5)	9 (0.8)	2.6 (0.8–8.1)	11 (1.3)	3.2 (1.0–10.1)
Total revisions	39 (2.4)		46 (3.4)		37 (3.3)		68 (8.3)	

Most revisions due to instability/dislocation occurred within the first 3 months.

The revisions in the fracture patients with uncemented stem were due to instability (n = 6), infection (n = 2) and periprosthetic fracture (n = 2), with a significant increased risk of revision due to instability compared with cemented stems (SHR 8.0, CI 2.7–23) (not shown in table). No uncemented stems in acute proximal humerus fractures were revised due to humeral stem loosening.

The revision causes differed to some degree between the brands (Table 8). Aequalis Reverse II and Aequalis Ascend Flex were revised mainly due to infections with SHR 4.8 (CI 2.0–11) and SHR 3.3 (CI 1.5–7.2), while SMR was revised due to instability, SHR 4.0 (CI 1.7–9.2).

**Table 8:** Reasons for revision in n (% revised) for the different brands of primary reverse shoulder arthroplasties by incidence and subhazard ratios (SHR). The most common reason for each brand is highlighted in bold. All other revision causes were merged and treated as 1 competing risk in the analyses. Results presented for the whole study period adjusted for age group, sex, fixation, previous surgery, and primary diagnosis. Delta Xtend was used as reference

Reason for revision	Delta Xtend n = 3,865	Aequalis n = 32	TESS n = 261	Promos n = 106	Aequalis Reversed II n = 190	SMR n = 242	Compre-hensive n = 392	Aequalis Asc Flex n = 369	JRI Vaios n = 37
Instability									
n (%)	47 (1.2)		2 (0.8)	4 (3.8)	2 (1.1)	14 (5.8)	1 (0.3)	8 (2.2)	1 (2.7)
SHR (CI)	1 (Ref.)	–	0.5 (0.1–2.3)	2.5 (0.7–8.4)	1.1 (0.2–4.4)	4.0 (1.7–9.2)	0.2 (0.1–2.1)	1.8 (0.7–4.3)	1.8 (0.2–15)
Infection									
n (%)	39 (1.0)	2 (6.3)	2 (0.8)	1 (0.9)	8 (4.2)		2 (0.5)	14 (3.8)	1 (2.7)
SHR (CI)	1 (Ref.)	3.1 (0.6–15)	0.4 (0.1–1.9)	0.8 (0.1–6.0)	4.8 (2.0–11.3)	–	0.7 (0.2–3.1)	3.3 (1.5–7.2)	2.7 (0.3–24)
Fracture									
n (%)	11 (0.3)		8 (3.1)	2 (1.9)		3 (1.2)	2 (0.5)	4 (1.1)	1 (2.7)
SHR (CI)	1 (Ref.)	–	14.2 (3.8–52)	8.2 (1.2–57)	–	11.2 (2.4–52)	3.4 (0.8–15)	8.7 (2.2–34)	14.3 (1.8–111)
Glenoid loosening									
n (%)	6 (0.2)		6 (2.3)				1 (0.3)	1 (0.3)	
SHR (CI)	1 (Ref.)	–	3.6 (0.9–14)	–	–	–	0.9 (0.1–8.0)	0.6 (0.1–5.7)	–
Humeral loosening									
n (%)	4 (0.1)			1 (0.9)	1 (0.5)				
SHR (CI)	1 (Ref.)	–	–	40.5 (5.3–310)	4.2 (0.4–47.2)	–	–	–	–
Other									
n (%)	29 (0.6)			1 (0.9)				5 (1.4)	1 (2.7)
SHR (CI)	1 (Ref.)	–	0.6 (0.1–6.0)	1.3 (0.2–12)	–	–	–	1.8 (0.4–7.7)	2.3 (0.5–36)
Total revisions									
n (%)	130 (3.4)	2 (6.3)	19 (7.3)	9 (8.5)	11 (5.8)	17 (7.0)	6 (1.5)	32 (8.7)	4 (11)

## Discussion

The primary aim of our study was to evaluate the survival of different RSA designs and brands reported to the NAR from 2007–2022, and factors associated with revision.

We found high survival of the most frequently used implants, and comparable risk of revision for RSAs with onlay and inlay designs, the latter being the most common design used in Norway. All brands in our study had more than 90% implant survival at 5 years, but an increased risk of revision was seen for the Aequalis (Reversed II and Ascend Flex) and SMR brands.

In accordance with previous studies, we found increased risk of revision in men and patients with fracture sequelae [5,11,12]. We did not find increased risk of revision in the younger age groups.

The reasons for revision in our cohort were similar to other series reporting on primary RSA [13,14] and also the review by Zumstein et al. [15], with instability and infection as the most frequent causes of revision.

Previous surgery in the same shoulder before a primary arthroplasty has been described as a risk factor for revision, especially revisions due to infections [16]. We found, however, an increased risk of revision only in acute fracture patients with previous surgery, but not in the fracture sequelae, rotator cuff, or osteoarthritis patients where we would expect previous surgeries to have an impact. Previous surgery in the fracture patients was in some cases fracture surgery (fractures of glenoid, of the greater tubercle, and proximal humeral shaft) indicating an osteoporotic population, while other surgeries were not likely related to the acute fracture diagnosis (subacromial decompression, biceps tenotomy, labral fixation).

Humeral stems can be either cemented or uncemented. Similar functional outcome has been reported [17], but differences in complication profile are described [17] and recommendations for fixation in different diagnoses are not established.

In most published studies, surgeons have applied cemented prostheses for acute proximal humerus fractures, but proponents for uncemented fixation argue that the cementless technique is faster, less expensive, has lower rates of complications, and an easier revision of the stem when necessary. A systematic review of reverse shoulder arthroplasty for acute proximal humerus fracture by Rossi et al. described similar functional results with the use of cemented and uncemented stems, and similar reoperation rates for both [18]. They found, however, a higher rate of complications in the uncemented cohort without any specifications of the complications. The study was limited by very few level 1 and 2 studies, low number of patients, short follow-up, and no control groups in many of the included studies. In our study, we included a high number of fracture patients and uncemented stems had more than 3 times increased risk of revision compared with cemented stems when applied for acute proximal humerus fractures. The revisions of uncemented stems were due to instability, infection, and fracture. These revision causes are comparable to the revision causes of uncemented hemiarthroplasties for femoral neck fractures [19]. The increased risk of revision due to instability could be caused by subsidence of the stem or malpositioning of the stem due to the fracture pattern and loss of stem stability.

Glenoid loosening was previously the main reason for revision [20]. However, modifications of the RSA technique have significantly reduced the failure rate [21]. This improvement is probably due to changed prosthesis design and focus on inferior glenoid placement, but presumably also a result of surgeons’ increased experience with the technique and implants in recent years. Glenoid loosening as a reason for revision in our study was seen in 15 patients (0.3%).

We found increased risk of revision due to periprosthetic fracture for the TESS, SMR, Aequalis Ascend, Promos, and JRI Vaios implants compared with Delta Xtend. However, our incidence of revision for fractures was lower than reported in a systematic review by Dolci [22].

Even if we found a statistically increased risk of revision for several brands in our material compared with the Delta Xtend both at 5-year follow-up and for the whole follow-up period, we cannot draw firm conclusions on differences in long-term survival due to low numbers left at risk and broad confidence intervals for most brands beyond 5 years’ follow-up.

The more recently introduced onlay prostheses have shown some biomechanical advantages and a supposedly better range of motion than traditional inlay-type implants [3].

A systematic review and meta-analysis by Larose et al. [23] found similar clinical improvements with the 2 designs. Less scapular notching but a higher rate of scapular spine fractures was reported for onlay implants, but revision rate was not reported. A recent report from the New Zealand joint registry compared inlay and onlay RSA and found higher mid-term survival for the inlay designs, but better functional results for the onlay designs [4]. The authors state, however, that the difference in functional result was statistically significant, but below the threshold for clinical significance. From the Australian registry a recent report showed increased risk of revision for inlay compared with onlay design [13], whereas in our study we found comparable risk of revision with the onlay designs compared with the traditional inlay designs.

Among the inlay designs the most frequent reason for revision was instability/dislocation according to our findings. The SMR (inlay) had the highest risk of revision due to instability. SMR is the only arthroplasty in our study with a medialized glenoid and humerus (MGMH) according to Werthel et al. [24]. For the onlay designs prostheses there were more revisions due to infection. Higher incidence of infection is not likely to be caused by the arthroplasty design, but rather patients, hospital, or surgeon factors. Hospital factors may also play a role in the overall rate of revision because some hospitals using the onlay design had an elective profile, and for fracture diagnosis most patients in our study received inlay design arthroplasties.

### Strengths

The primary strength of this study is the high number of arthroplasties included and the long follow-up time. The completeness of reporting to the NAR is high and has been stable over time [25], and all hospitals in Norway report to the register. This enables evaluation of rare reasons for revision that would otherwise be impossible to assess at a single institution.

### Limitations

First, the reasons for revision are based on the surgeons’ report only. We suspect that some of the unknown reasons for revision and those revised because of pain alone may in fact be low-grade infections that were not suspected at the time of surgery due to the lack of clinical manifestations of infection. Second, the number of revisions is underestimated since the completeness of reporting of revisions is only 85% [11]. Third, patient-reported outcomes (PROMs) have just recently been added to the registration in the NAR. Accordingly, no PROMs were available for this study.

Fourth, since osteosynthesis of periprosthetic fractures where the prosthesis is not revised was not included in our analysis, these fractures are likely underestimated. There is no reason to suspect this underestimation to be different between the implants.

Fifth, hospital and surgeon volume may also play a role in the risk of revision [26]. Several of the hospitals in our study performed less than 10 shoulder arthroplasties annually and this could make it difficult to generalize the results of arthroplasties performed at many small hospitals. In addition to this, some of the implant brands included in the study had short follow-up.

Sixth, even if the number of arthroplasties is high, the number of revisions is a limitation when it comes to analyzing the different reasons for revision and some of the SHRs are based on very low numbers and display wide confidence intervals. Therefore, the evaluation of revisions on brand level, or on different diagnoses, must be done with caution.

Seventh, in this study we did not consider different glenoid sizes. Smaller glenosphere sizes have previously been described as a risk factor for instability and risk of early revision [14].

Eighth, we classified the prostheses as either inlay or onlay, but we did not take into consideration other design features that can alter the lateralization of the arthroplasty, such as lateralization of the baseplate or glenosphere or altering of the neck shaft angle.

### Conclusion

We found 10-year survival of the most common implant; Delta Xtend, was 95%. Some prosthesis brands had higher rates of revision than the most frequently used implant, but these differences could be influenced by low number of cases/surgeons/hospitals. Factors that were associated with an increased risk of revision were male sex, fracture sequelae diagnosis, and uncemented humeral stem in acute fracture patients.

In perspective, surgeons should be aware of the risk of revision due to instability, especially in fracture sequelae patients. Cemented stems should be preferred when treating acute proximal humeral fractures.

## Figures and Tables

**Figure:**
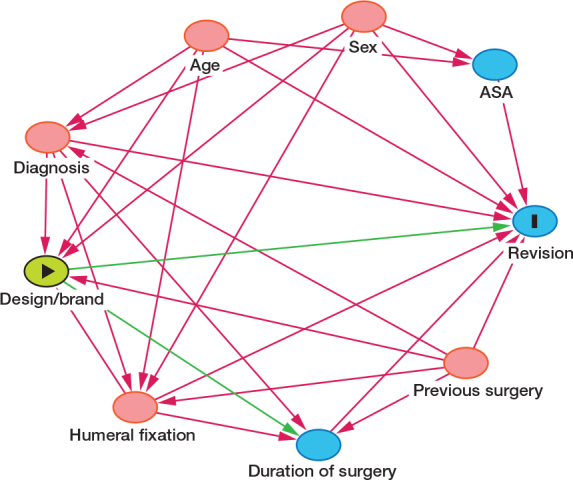
DAG diagram for the selection of variables with design/brand as exposure.

**Figure:**
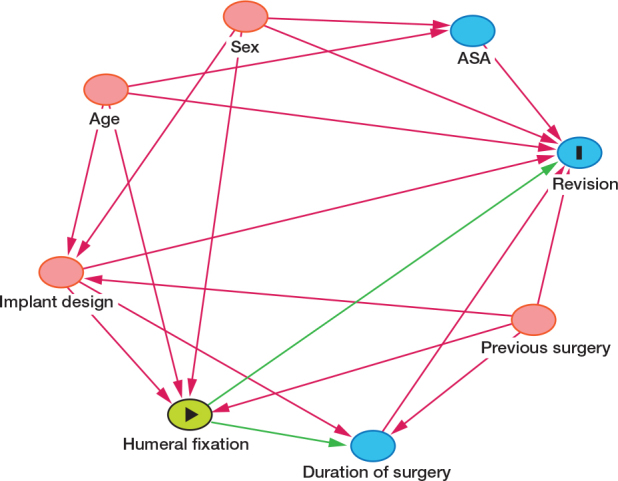
DAG diagram for the selection of variables for analyses of each diagnosis separately with humeral stem fixation as exposure.

## Notes

RMH, AMF, JEG, GH and ONF planned the study. RMH wrote the initial draft of the manuscript. AMF and RMH performed the statistical analyses. All authors contributed to the interpretation of the findings and critical revision of the manuscript through all stages of the study.

The authors thank the Norwegian surgeons for their contribution by thoroughly reporting to the NAR.

Handling co-editors: Jeppe Vejlgaard Rasmussen and Philippe Wagner

*Acta* thanks Ville Aarimaa and Theis Muncholm Thillemann for help with peer review of this manuscript.
